# Editorial: Unsolved challenges in hepatitis B and hepatitis C: from prevention to treatment

**DOI:** 10.3389/fcimb.2023.1321432

**Published:** 2023-10-25

**Authors:** Ming Yue

**Affiliations:** Department of Infectious Diseases, The First Affiliated Hospital with Nanjing Medical University, Jiangsu Province Hospital, Nanjing, Jiangsu, China

**Keywords:** hepatitis B virus, hepatitis C virus, host, prevention, treatment

Currently, hundreds of millions of people worldwide are infected with hepatitis B virus (HBV) or hepatitis C virus (HCV). End-stage liver diseases caused by these viruses claim countless lives every day. Existing hepatitis B vaccine have effectively controlled the transmission of hepatitis B. However, there is currently no vaccine available for HCV. Over the past 10 years, in terms of hepatitis B treatment, the combination of nucleoside analogs (NAs) with pegylated interferon α-2b has significantly improved the clinical efficacy in the treatment of hepatitis B, and has even achieved clinical cure in some patients; as for hepatitis C, the approval of oral interferon-free direct-acting antivirals (DAAs) revolutionized the therapy of chronic hepatitis C infection and achieved the cure of hepatitis C in many patients. However, the rates of awareness, diagnosis and treatment for these two viral hepatitis are still suboptimal. In addition, what cannot be ignored is, the different genetic backgrounds of the host and the genetic diversity of viruses driven by genomic mutations may also lead to the clinical uncertainties in some patients. Therefore, there are still many unsolved challenges to achieve the WHO “eliminate hepatitis” goal, and we should not give up our attention and exploration.

The Research Topic: Unsolved challenges in hepatitis B and hepatitis C: From Prevention to Treatment of Journal: Frontiers in Cellular and Infection Microbiology focus on the new research findings made in these fields in recent years. This editorial will summarize and discuss these developments.

In the context of hepatitis B and C prevention, Meyer et al. conducted an assessment of prescription numbers for DAA medications within Germany during the COVID-19 pandemic. Their findings revealed a significant overall decrease in prescriptions, indicating a temporary gap in hepatitis C treatment. This highlights the need for hospitals and private clinics to adapt more quickly in future pandemics to ensure continued access to care. Furthermore, political strategies should place greater emphasis on maintaining essential healthcare services during periods of restricted access due to infectious disease outbreaks. Ndunguru et al. found that conducting promotional activities and mobilizing resources at primary healthcare facilities in Tanzania is crucial to promote hepatitis B vaccination.

In the field of epidemiological research, Qian et al. quantitatively analyzed the long-term spatiotemporal heterogeneity of hepatitis B and C incidence in China from 2010 to 2018, as well as the impact of socio-economic factors on their risk using Bayesian spatiotemporal hierarchical models. The results indicate that there is significant spatial and temporal heterogeneity in the risk of hepatitis B and C. These findings contribute to the effective allocation of resources and the design of intervention measures to combat viral hepatitis. Another research conducted by the Jia et al., utilizing Bayesian analysis, SNP genotyping, and sequencing methods, revealed that HCV 3b subtype is the predominant circulating strain in Yunnan Province, China and the prevalence of HCV 3a and 3b subtypes is rapidly increasing. He et al. found that the IFNL4 gene, along with the MxA and MxB genes, is associated with HCV infection in the Yunnan population or the liver function of HCV patients. These findings underscore the need for the implementation of more stringent public health measures for prevention and treatment to curb the spread of the virus.

In the context of auxiliary diagnosis and model prediction of hepatitis B and hepatitis C, Li S. et al. developed and validated a non-invasive model that includes parameters such as aspartate aminotransferase (AST), hepatitis B e-antigen (HBeAg), and platelet. This model aimed to identify pseudo-immune tolerance in chronic hepatitis B (CHB) patients by predicting significant liver fibrosis, thereby assisting in the formulation of more appropriate treatment strategies. Zhao et al. reported a correlation between serum hepatitis B surface antigen (HBsAg) and hepatitis B core-related antigen (HBcrAg) levels and inflammation grading in HBeAg-positive CHB patients before nucleos(t)ide analogues (NAs) therapy. Furthermore, the combination of HBsAg and AST showed excellent diagnostic capability for significant inflammation. These non-invasive biomarkers contribute to the diagnosis and grading of liver necroinflammation. Zhang Q. et al. established an effective individualized nomogram, including clinical and endoscopic features (such as the size of varices, red wale marks, ascites, spleen thickness, γ- glutamyltransferase, and hematocrit), to predict the risk of first variceal hemorrhage in HBV-related gastroesophageal varices patients. This can assist clinicians in formulating more appropriate prevention strategies. Wang et al. assessed the performance of pregenomic RNA (pgRNA) and HBcrAg kinetic in predicting HBeAg seroconversion and HBsAg reduction postpartum in HBeAg-positive pregnant women. They found that in chronic HBV carriers who were HBeAg-positive pregnant women receiving antiviral prophylaxis, a postpartum decrease in pgRNA and peak ALT levels helped identify patients with HBsAg reduction after treatment cessation.

In terms of hepatitis B treatment, Li J. et al. in a prospective multicenter study, found that factors associated with persistent HBV DNA positivity in patients with chronic hepatitis B treated with entecavir included high HBV DNA levels, low anti-HBc levels, and HBeAg serum positivity. However, persistent viremia patients had a lower rate of fibrosis progression and risk of developing HCC. Guo et al. found that in patients who achieved pegylated interferon-induced HBsAg loss who achieved functional cure with HBsAg loss, those who had both HBeAg negativity and higher anti-HBs levels at the end of PEG-IFN treatment had the low risk of HBsAg reversion after PEG-IFN discontinuation. Li M. et al. found that antiviral therapy could reverse decompensation of ascites in HBV-related first decompensated cirrhosis, and ALT and HBV DNA levels were associated with ascites recompensation. Cai et al. established a convenient *in vitro* cell model for screening compounds that target elongation factor Tu GTP-binding domain containing 2 (EFTUD2) as antiviral agents against hepatitis B. In this model, it was confirmed that small-molecule compounds, plerixafor and resatorvid, can inhibit HBV by upregulating the EFTUD2. This provides a new avenue for the development of novel anti-hepatitis B drugs that target host factors rather than viral enzymes.

HBV and HCV infections may also have some extrahepatic effects and can potentially co-infect with the human immunodeficiency virus (HIV). Tao et al. collected hepatitis test results and bone mineral density (BMD) from respondents in the NHANES database and compared BMD between respondents who were positive and negative for respondents related to hepatitis B and C. The results of multiple regression analysis revealed that positive tests for HBsAg and hepatitis C RNA were associated with a reduction in BMD. Positive serology for these hepatitis indicators may increase the risk of reduced BMD. Zaltron et al. reported a case of occult HBV infection (OBI) reactivation in a HIV/HCV co-infected patient who was lost to follow-up after DAAs treatment. Upon re-encounter with the patient, the individual exhibited high levels of plasma HIV-RNA, severe immunosuppression. After reintroducing antiretroviral treatment, an immune reconstitution inflammatory syndrome (IRIS) was diagnosed, along with high level of HBV-DNA load and transaminase. This case report highlights the dynamic balance between the virus and the host immune system, emphasizing the importance of strict monitoring of HBV serological and virological markers for patients with compromised immune systems receiving tenofovir or lamivudine-sparing regimens, even in the absence of a hepatitis flare. Zhang Q. et al. conducted a retrospective cohort study to investigate the clearance rate of HBsAg in Chinese HIV/HBV co-infected patients on long-term tenofovir disoproxil fumarate-containing antiretroviral therapy and found that advanced age, high CD4 cell count, and positive HBeAg at baseline could be regarded as potential predictors and biological markers for HBsAg clearance in patients with HIV/HBV coinfection.

This Research Topic also includes valuable reviews. CD73 is fundamentally an enzyme and a crucial component of the adenosine signaling pathway. It mediates the conversion of inflammatory ATP into the immunosuppressant adenosine, dynamically regulating processes such as hepatic steatosis, inflammation, and fibrosis in the liver. Shi et al. provided a comprehensive review of the close connection between CD73-mediated adenosine metabolism and the liver, as well as its role in the pathogenesis of various liver diseases. Functional cure for hepatitis B, characterized by the absence of detectable HBsAg in the patient’s serum and HBV DNA, remains a rarity, achievable only in a fortunate few. The difficulty in achieving a cure for hepatitis B primarily stems from covalently closed circular DNA (cccDNA) of HBV, integrated HBV DNA, high viral loads, and compromised host immune responses. In the pursuit of developing more effective treatments and achieving higher rates of functional cure, the industry continues relentless efforts in the development of innovative drugs. Pan et al. have summarized the functions and mechanisms of various synthetic molecules, natural products, and traditional herbal remedies and discussed therapeutic strategies for modulating the host immune response. DExD/H-box helicases play crucial roles in various biological processes, including hematopoiesis, cell proliferation, metabolism, signal transduction, immune responses, and inflammation. They can sense non-self viral nucleic acids and participate in regulating multiple antiviral immune signaling pathways, including Toll-like receptor and retinoic acid-inducible gene I-like receptor pathways. You et al. reviewed the current understanding of the effects of different DExD/H-box helicases on HBV replication regulation, the role of HBV in altering DExD/H-box helicases, and the potential of targeting DEAD/H-box helicase to eliminate HBV infection. Further understanding of the effects of DExD/H-box helicase on HBV infection may aid in the eliminate hepatitis B. 308 words.

In summary, this Research Topic encompasses the latest advancements in the prevention, diagnosis, and treatment of hepatitis B and hepatitis C. These developments provide a theoretical foundation and scientific basis for efforts aimed at preventing and ultimately eradicating these two viral liver diseases in the future.

## Author contributions

MY: Funding acquisition, Writing – original draft, Writing – review & editing.

